# Understanding Self-Restraint in Neurodevelopmental Conditions: A Primer for Assessment and Treatment

**DOI:** 10.3390/bs16010060

**Published:** 2025-12-30

**Authors:** Kayla Mann, John Michael Falligant

**Affiliations:** Department of Psychological Sciences, Auburn University, Auburn, AL 36849, USA

**Keywords:** autism, assessment, functional analysis, self-restraint, self-injury

## Abstract

Self-restraint, characterized by self-initiated restriction of movement (e.g., intertwining limbs, sitting on hands), is most commonly observed in individuals with neurodevelopmental conditions who also engage in self-injurious behavior (SIB). These behaviors may serve to prevent SIB but can also cause injury and interfere with everyday functioning. Findings from past research suggest that self-restraint encompasses a heterogeneous class of behaviors and may serve multiple operant functions. We review conceptual models and empirical studies of the structural and functional dimensions of self-restraint, including procedures for identifying controlling contingencies and reducing the occurrence or impact of self-restraint on daily life. Available interventions, such as noncontingent reinforcement, differential reinforcement, functional communication training, and restraint fading, are discussed in the context of their limitations and successes. We conclude with recommendations for future research aimed at clarifying the functional properties of self-restraint and developing systematic approaches to its assessment and treatment.

## 1. Introduction

Self-restraint is a functional class of behavior characterized by self-initiated restriction of movement, typically occurring among individuals with neurodevelopmental conditions and neurogenetic syndromes (Marlow et al., 2024). Extreme and prolonged self-restraint poses risks to physical health, including halted motor development, muscular atrophy, decreased circulation, and functional immobility (Isley et al., 1991; Smith et al., 1992). Chronic self-restraint also has the potential to severely limit adaptive behavior (e.g., self-feeding, engagement with academic and leisure materials) and opportunities for community inclusion. Although self-restraint can have profound implications for health, independence, and quality of life, it remains one of the least understood and least studied behaviors observed in individuals with neurodevelopmental conditions. Given the substantial gaps in the literature and the practical challenges this behavior presents to clinicians, a contemporary primer is needed to synthesize existing knowledge and identify directions for future research.

## 2. Phenomenology

### 2.1. Prevalence

Self-restraint is more commonly observed in individuals with neurodevelopmental conditions than in their neurotypical peers^1^, and it is especially prevalent among individuals with severe or profound disabilities (Fovel et al., 1989). Additionally, research indicates that self-restraint occurs disproportionately among individuals who also engage in self-injurious behavior (SIB; Fisher & Iwata, 1996; Richards et al., 2017; Smith et al., 1992). Prevalence estimates for self-restraint vary considerably across studies. Investigations have reported a wide range of prevalence rates, including 2.9% (Fovel et al., 1989), 20.5% (Hagopian et al., 2015), 34% (Marlow et al., 2024), 46% (Powell et al., 1996), 64% (Hyman et al., 2002) and 76.1% (Oliver et al., 2003). Findings regarding demographic correlates such as age and gender are inconsistent. For instance, Richards et al. (2017) reported comparable prevalence of self-restraint among children (40.9%) and adults (42.6%), whereas earlier studies suggested higher rates among younger individuals (Fovel et al., 1989). While Hyman et al. (2002) and Marlow et al. (2024) found no differences in prevalence by gender, Oliver et al. (2003) found males were significantly more likely to engage in self-restraint than females. Such discrepancies underscore the substantial heterogeneity in the occurrence of self-restraint, which may be attributable to differences in sampling methods, participant characteristics, operational definitions, and assessment procedures (see below).

### 2.2. Structural Dimensions

Analysis of self-restraint is complicated by its heterogeneity, as it can present in a wide range of topographies (Schroeder & Luiselli, 1992). Some evidence suggests that certain forms—such as holding onto other people or their clothing—may be more common among younger individuals (Richards et al., 2017), but more research is needed to understand how age, developmental level, and other characteristics contribute to the expression of specific self-restraint behaviors.

In an early survey on self-restraint and challenging behavior, Isley et al. (1991) proposed that self-restraint could be grouped into three broad categories. The first includes behaviors in which movement is restricted through the use of inanimate objects, such as wrapping limbs in clothing or entangling them with other materials. The second involves the use of one body part to restrict another—such as clasping the hands together, sitting on one’s hands, or lying on one’s arms. The third category consists of behaviors that do not directly restrict movement but result in restraint, such as signaling or requesting the application of protective equipment or mechanical restraints. More recently, Oliver et al. (2003) defined self-restraint according to 23 distinct topographies, which they grouped into four subscales based on the means by which restraint was achieved. The first subscale, use of clothing or materials (9 items), included behaviors such as entwining hands in clothes, putting arms or hands into sleeves or trousers, placing hands in pockets, and wrapping material around the arms or body. The second subscale, use of the body (9 items), encompassed topographies such as lying on the stomach with hands behind the back, sitting on hands, clasping hands behind the neck^2^, placing hands between the legs, folding arms, or intertwining arms or fingers. The third subscale, use of objects (3 items), captured behaviors like entwining arms in furniture, placing hands in tight spaces, or carrying a specific object continuously. The fourth and final subscale, use of other people (2 items), included behaviors such as requesting to have one’s hands or arms held by another person.

In one of the few studies to examine the prevalence of different topographies of self-restraint, Powell et al. (1996) reported that, among a sample of 46 individuals residing in a state institution, the most common form of self-restraint was holding or squeezing objects—a topography exhibited by 50% of these individuals. Other common forms included holding onto other people or their clothing (41.3%) and positioning oneself in a way that restricts movement (39.1%). The majority of individuals in this sample engaged in a single form of self-restraint (52%), whereas a smaller proportion exhibited two or more distinct forms. These findings align with more recent data reported by Marlow et al. (2024), who also identified holding or squeezing objects (32%) and holding onto others or their clothing (32%) as the most prevalent self-restraint topographies. Asking for hands to be held was also common (30%), whereas choosing mechanical restraint was the least frequently observed (1%).

### 2.3. Functional Dimensions

Self-restraint appears to interact closely with SIB, but the specific form of their relationship varies and is not fully understood. Several functional relations may exist between SIB and self-restraint (Fisher & Iwata, 1996).

#### 2.3.1. Positive Reinforcement

SIB and self-restraint may be members of the same functional response class maintained by social positive reinforcement. For example, both behaviors may be maintained by social consequences in the form of a reprimand (e.g., “Stop doing that!”) or praise contingent upon a response that is incompatible with SIB (e.g., “Great job having safe hands”; Derby et al., 1996). Or, alternatively, access to self-restraint may positively reinforce self-injury. Smith et al. (1996) examined this hypothesis by evaluating rates of SIB (i.e., head hitting and hair pulling) and self-restraint (i.e., entangling hands in articles of clothing) exhibited by a 32-year-old woman diagnosed with profound retardation and blindness. Rates of SIB were compared across various conditions in which access to self-restraint was either: continuously available, presented as a consequence for SIB, or unavailable (i.e., any attempts to self-restrain were blocked). The results suggested that the individual’s SIB increased when access to restraint was contingent upon SIB and decreased when restraint was unavailable, thereby supporting the positive reinforcement hypothesis. This pattern is commonly observed in the natural environment, where caregivers may allow or even encourage self-restraint following instances of SIB.

#### 2.3.2. Negative Reinforcement

SIB and self-restraint may be members of the same functional response class maintained by social negative reinforcement, such as avoidance or escape from demands. Given that it is often laborious to block or prevent self-restraint and SIB, it is possible that caregivers might present fewer or no demands to individuals who engage in these responses (Smith et al., 1996). A more prominent view specifically holds that self-restraining behaviors lessen the aversive consequences of self-injurious episodes by delaying or terminating SIB. This is arguably the most widely discussed theory as to why self-restraint occurs (e.g., Baroff & Tate, 1968; Fisher et al., 1996; Rojahn et al., 1978; Scheithauer et al., 2015; Silverman et al., 1984). Studies supporting this idea generally find that self-restraint and SIB are negatively correlated, meaning the provision of protective equipment or unrestricted access to self-restraint tends to significantly reduce or eliminate SIB (e.g., Silverman et al., 1984). Notably, individuals who engage in both self-restraint and self-injury are more likely to display other compulsive behaviors (Hyman et al., 2002; Muehlmann & Lewis, 2012; Powell et al., 1996). These data lend support to accounts that conceptualize SIB as a nonfunctional and possibly involuntary^3^ response arising under weak inhibitory control, with self-restraint functioning as an adaptive though maladaptive form of response suppression (e.g., Gedye, 1992).

Physiological processes may also contribute to the relation between SIB and self-restraint. For some individuals, self-directed behaviors may modulate autonomic arousal. Barrera et al. (2007), for example, reported that blocking access to preferred restraint materials for a participant (Tammy) produced sharp increases in heart rate, indicating heightened physiological stress when self-restraint was unavailable. These findings raise the possibility that self-restraint may, in some cases, serve as a proactive response to reduce arousal and prevent escalation to SIB.

It is also possible that self-restraint and SIB are functionally independent and operate under separate contingencies of reinforcement (e.g., both positive and negative). In one of the few empirical demonstrations of this account, Rapp and Miltenberger (2000) described an 11-year-old boy who reliably engaged in both self-restraint and SIB simultaneously. The participant engaged in SIB in the form of hand-to-head slapping with one hand and self-restraint in the form of wrapping his other hand inside his shirt or shoelaces. The results from a standard functional analysis did not indicate a specific function of SIB for this participant, however a subsequent pairwise analysis showed that SIB was occasioned by the removal of a tangible item. On the other hand, high, undifferentiated levels of self-restraint were observed across all conditions in the functional analysis. An analysis of self-restraint was also conducted to further clarify the function of self-restraint and evaluate its relationship with SIB. In this assessment, levels of SIB and restraint were compared during both play and no-play conditions. The possibility that SIB was occurring to gain access to self-restraint was ruled out in a contingent restraint condition, where therapists would block access to self-restraint unless SIB occurred. Near-zero rates of self-restraint were displayed during the contingent restraint condition. The purpose of the no-play conditions was to assess the extent to which low environmental stimulation would increase the reinforcing efficacy of self-restraint. Results supported this hypothesis, as the no-play context produced higher levels of self-restraint than the play context. Therefore, researchers concluded that the participant’s SIB was maintained by access to tangibles while his self-restraint was maintained by automatic positive reinforcement.

## 3. Assessment

Assessment of self-restraint can proceed along two complementary lines. Structural assessments describe the form and characteristics of the behavior (e.g., developing a topography profile, identifying materials or postures involved), whereas functional assessments evaluate the controlling variables (e.g., whether access to restraint suppresses SIB, serves as reinforcement, etc.). Combined, these approaches provide an empirical foundation for hypothesis development and subsequent treatment planning.

### 3.1. Questionnaires

Powell et al. (1996) developed the Self-Injury/Self-Restraint Checklist (SRC), which is completed simply by indicating any item exhibited by the subject that was repetitive, self-initiated, and appeared to prevent or stop movement of body parts (Powell et al., 1996). Stakeholders endorse the presence/absence of specific behaviors across recent observation periods, generating a profile of restraint forms (e.g., use of clothing, body positioning, objects, or other people). More recently, Oliver et al. (2003) developed the Self-Restraint Questionnaire (SRQ). This assessment is very similar to the SRC and includes 23 items, each describing a single topography of self-restraint (e.g., “Does the individual entwine his/her hands in the front part of the clothes that he/she is wearing?”), accompanied by an illustration of each topography. A relative or care worker who knew the participant well completed the questionnaires and was instructed to approximate the frequency of each behavior’s occurrence over the last month using a five-point scale (with 0 being “never occurred” and 4 being “occurred all the time”). Results showed that approximately 76% of the individuals who participated in the study engaged in at least one form of self-restraint, with “asking for their hands to be held” being the most commonly observed. A notable finding from this study indicated that the prevalence of self-restraint was significantly higher in participants who did not wear protective devices (e.g., arm splints, gloves, helmets) than in those who did. Inter-rater reliability of the SRQ was examined, and acceptable levels were seen across the majority of items. Validity was also assessed by directly observing 14 individuals during three 30-min sessions across the day and comparing agreement between items endorsed by caregivers on the SRQ and the presence of various self-restraint topographies observed. Both methods have been shown to be valid and reliable in identifying specific and discrete topographies of self-restraint behaviors. However, functional assessment is critical for understanding why self-restraint occurs and how to best intervene.

### 3.2. Functional Analysis

Functional analysis (FA) is both a process for identifying the controlling variables of challenging behavior (e.g., SIB, aggression, self-restraint) and a methodology for experimentally^4^ examining those variables in a systematic manner. A reasonable approach, reflected in the clinical literature, is to evaluate both SIB and self-restraint and, as needed, introduce available/blocked and contingent/noncontingent access conditions to isolate the role of self-restraint relative to SIB. An example of this general approach is found in early work by Smith et al. (1992), who investigated the functional properties of self-restraint and SIB using a variation of procedures developed for the functional analysis of SIB (Iwata et al., 1994). The participants experienced the four primary functional analysis conditions (attention, demand, alone, and play), arranged in a multielement format. However, in the present experiment, each of these assessment conditions was conducted under two sub-conditions to control for the effects of self-restraint: restraint available and restraint unavailable. In “restraint available” conditions, participants had free access to their typical form of self-restraint, including any materials or items used to facilitate restraint, and no programmed consequences were provided for engaging in self-restraint. In “restraint unavailable” conditions, restraint materials were not available to the participant. Findings indicate that self-restraint, akin to SIB, is functionally heterogeneous across individuals. Two of the participants engaged in self-restraint almost continuously across all sessions in which it was available. SIB rarely, if ever, occurred during these times when restraint was available. Meanwhile, during the conditions in which restraint was not available, the rates of SIB increased significantly. These results are most supportive of the hypothesis that self-restraint functions as an avoidance response to prevent the aversive effects of SIB.

More recently, Scheithauer et al. (2015) extended the procedures used by Smith et al. (1992) to evaluate whether a participant’s self-restraint was maintained by negative reinforcement. The participant was a 12-year-old girl who engaged in hand-to-head SIB and self-restraint in the form of sitting on her hands or placing her arms between her folded legs. Three functional analyses of SIB were conducted. The purpose of the first two FAs was to manipulate the availability of self-restraint and observe the effects on SIB when self-restraint was available versus unavailable. In the final FA, SIB was blocked using arm splints, and the corresponding rate of self-restraint was observed. Blocking SIB presumably removed any aversive consequences produced by SIB. Results showed that self-restraint decreased when SIB was blocked, further supporting the idea that self-restraint was maintained by negative reinforcement.

While the previous FA results are representative of the negative reinforcement hypothesis, highly idiosyncratic relationships between SIB and self-restraint have been documented in the literature. For instance, one participant in the Smith et al. (1992) study produced data showing that both rates of SIB and rates of self-restraint were highest in the demand condition of the FA. These results are most consistent with the hypothesis that self-restraint serves a similar function to SIB, in this case, escape from task demands. Interestingly, a different participant in this study displayed undifferentiated rates of SIB across FA conditions, while his rate of self-restraint gradually decreased to near-zero levels during the course of assessment. These results suggested entirely different functions for the two behaviors (SIB seemed to be automatically maintained while self-restraint was more likely maintained by a socially mediated consequence). The highly variable results across participants in Smith et al. (1992) exemplify the complex functional relationships that may exist between SIB and self-restraint.

Vollmer and Vorndran (1998) built on previous work by employing a modified functional analysis to further evaluate the relationship between SIB and self-restraint. In this study, the test versus control comparison of interest was access to restraint materials contingent on SIB versus noncontingent access to restraint materials during leisure and alone conditions. The results showed that SIB rates were elevated when access to restraint materials was made contingent on the behavior, compared to low rates in both control conditions. Rooker and Roscoe (2005) found similar outcomes in their functional analysis which included the following conditions: (1) noncontingent access to self-restraint, (2) contingent access to self-restraint, and (3) contingent access to self-restraint plus noncontingent access to alternative restraint. The purpose of this was to evaluate whether SIB was maintained by access to self-restraint and to evaluate whether the suppressive effects of continuous access to self-restraint materials generalized across items. They found that SIB only occurred during the contingent access to restraint condition. The findings in both of these studies support the hypothesis that access to self-restraint materials can reinforce SIB. Collectively, these and other studies provide empirical support that self-restraint may serve different functions across (Fisher & Iwata, 1996) and within (Rapp & Miltenberger, 2000) individuals.

### 3.3. Pre-Treatment Assessments

#### 3.3.1. Competing Stimulus Assessments

In the Rooker and Roscoe (2005) study noted above, therapists conducted a paired choice preference assessment (PSPA) with nine candidate self-restraint items to identify the most preferred and effective forms of self-restraint for inclusion in their functional analysis. The items that were selected as most effective were ones which resulted in the highest levels of self-restraint and the lowest levels of SIB. They subsequently conducted a modified competing stimulus assessment, in which the same nine items were individually presented to the participant across four 3-min demand sessions. Two high-preferred items were identified in the PSPA (items approached on over 80% of the trials). Those same two items resulted in 100% engagement in self-restraint across sessions and no SIB in the competing items assessment, in contrast to the other seven items in which rates of SIB were elevated. The results of this assessment suggest that providing continuous access to preferred restraint materials may serve as an initial treatment option for individuals who exhibit SIB maintained by escape and by contingent access to self-restraint. Importantly, the items selected in the assessments were also deemed more socially acceptable alternatives to the existing forms of self-restraint (i.e., airplane pillow rather than a life vest), highlighting the utility of this approach for identifying safer substitutes when certain topographies of self-restraint are not consistently available or are otherwise prohibitive.

#### 3.3.2. Augmented-Competing Stimulus Assessments

Building on the approach described above, Hagopian et al. (2020) evaluated an augmented-competing stimulus assessment (A-CSA) with participants who engaged in treatment-resistant subtypes of automatically maintained challenging behavior, including two individuals who displayed self-restraint. The A-CSA is a pretreatment assessment designed to identify stimuli that are associated with reductions in challenging behavior, presumably through reinforcer competition or substitution. When access to items alone did not produce meaningful reductions in challenging behavior, the assessment incorporated additional test conditions, such as prompted engagement or response blocking. These procedures facilitated the identification of leisure items associated with high levels of engagement and low levels of both self-restraint and SIB. For some stimuli, these effects persisted even after prompting and blocking were withdrawn. Frank-Crawford et al. (2026) expanded on this work by conducting an A-CSA to identify and establish self-restraint items associated with reductions in the existing form of self-restraint and SIB. Their procedures differed from those used by Rooker and Roscoe (2005), who examined only the effects of items on SIB without measuring self-restraint. Frank-Crawford et al. also incorporated assessment conditions that used response promotion and disruption strategies to increase or maintain high levels of engagement with leisure items. Alternative self-restraint items included in the A-CSA were selected based on client preference, caregiver acceptability, the potential to compete with the existing form of self-restraint, and the likelihood of interfering with adaptive behavior (e.g., toy engagement). The study successfully identified at least one alternative self-restraint item that did not interfere with adaptive behavior for four participants. These items effectively replaced the participants’ existing forms of self-restraint without increasing rates of self-injury.

#### 3.3.3. Alternative Self-Restraint Assessment

Dawson et al. (2025) combined the use of multiple pre-treatment assessments for a young boy who engaged in multiple topographies of SIB and self-restraint. The assessments included a functional analysis, a reinforcer assessment, an alternative self-restraint assessment (ASRA), and a modified version of the A-CSA. Results from the functional analysis indicated that the participant’s SIB was maintained by access to self-restraint as it occurred at high levels when self-restraint was blocked but decreased when access to self-restraint was provided. The reinforcer assessment identified three stimuli that were associated with high rates of the target response (e.g., an eye gaze and card touch) and were selected due to the practicality of their delivery and endorsement by caregivers. The purpose of the ASRA was to identify less restrictive and more socially acceptable forms of self-restraint that could replace the child’s existing strategies (e.g., lying prone with hands under his stomach or entangling his shirt around his hands). In the ASRA, guided exposure to each alternative form of self-restraint was conducted by physically guiding the participant to engage in the response, and then subsequent sessions began in which no prompting or consequences were provided. Clinicians recorded engagement with each item/response and the rate of SIB across two-minute sessions for each item. Based on the results of this assessment, as well as caregiver input regarding accessibility, comfort, and perceived stigma, two alternative self-restraint responses were selected: placing hands behind the back and placing hands in a hand warmer. Finally, the A-CSA in the current study differed from the procedures described by Hagopian et al. (2020) in that the free access condition was omitted to mitigate the risk of injury produced by SIB. A response disruption and response promotion and disruption condition were successful in identifying items that competed with SIB. The combined results of the four assessments informed a unique reinforcement-based treatment package that reduced both the frequency of SIB and the reliance on mechanical restraints and restrictive self-restraint.

## 4. Treatment

When self-restraint and self-injury belong to the same functional response class, a single function-matched treatment may be effective for both. For example, Derby et al. (1996) used a reversal design comparing (a) noncontingent attention (NCR), (b) attention contingent on SIB, and (c) attention contingent on self-restraint. Contingent attention increased SIB while self-restraint remained low; contingent attention for self-restraint increased both responses; and NCR produced near-zero levels of both. Such findings, for example, might suggest that NCR is effective when attention maintains both responses. In practice, however, the functions of SIB and self-restraint often diverge or are difficult to resolve, and treatment therefore typically relies on multicomponent packages integrating reinforcement-based procedures, response blocking, systematic restraint fading, and use of protective equipment. For example, in the aforementioned study by Dawson et al., the multicomponent package comprised: (a) differential reinforcement of alternative behavior (DRA), (b) continuous access to competing stimuli and a preselected alternative self-restraint response, and (c) response blocking with redirection to the alternative self-restraint contingent on attempted SIB. In the DRA component, reinforcement was delivered for compliance with or tolerance of nonpreferred tasks, and was systematically thinned to more feasible and naturalistic levels across multiple contexts, with concurrent reductions in SIB and reliance on mechanical restraints.

The majority of peer-reviewed studies which have reported on interventions to reduce self-restraint have focused on specific topographies of self-restraint that involve the use of mechanical restraints or objects (e.g., arm splints, helmets, lap belts). Pace et al. (1986) demonstrated the utility of a stimulus fading approach to reduce restraint use with two adolescents with profound disabilities. The first participant required multiple types of protective equipment (e.g., wrist restraints tied to a belt, a large collar worn around the neck, and a helmet with plastic face guard) to reduce his self-injury. The study focused on one specific form of self-restraint used by this participant 24 h a day. He held rigid tubes extending from the shoulders to the hands, which prevented arm flexion and made hand biting impossible. Prompting and differential reinforcement procedures were implemented in which the experimenter prompted the removal of the restraints and provided praise contingent on the absence of restraint for a specified duration. These procedures successfully decreased self-restraint to near-zero levels but were associated with increases in SIB. Restraint fading was then implemented by gradually reducing the length of the arm tubes over a 22-day period and eventually replacing them with cloth tennis wristbands. Contingent reinforcement was also provided for the absence of SIB during this process. This combination of procedures was effective in that the wristbands acquired stimulus control over SIB and were far less restrictive than the previous form of restraint, allowing the participant to engage in self-help and academic skills.

There are some cases in which individuals engage in forms of self-restraint that cannot be easily faded. For example, a second participant in the Pace et al. study engaged in several forms of self-restraint, including putting his hands in his pockets, inside of his pants, under his thighs, and behind his back. He was also frequently restrained in rigid elbow splints that completely immobilized his arms. These various forms of restraint were associated with decreases in his SIB (e.g., vigorously scratching behind his ears) but resulted in severely limited use of his hands and arms. In this case, clinicians eliminated both SIB and self-restraint by modifying the restraint form, introducing pneumatic air splints that could be more easily faded. Fading involved gradually reducing the air pressure within the splints, while reinforcement was delivered contingent on toy contact and instances of SIB were placed on extinction. The results indicated that the participant gained complete mobility of his arms and demonstrated substantial increases in adaptive behaviors. In summary, Pace et al. (1986) demonstrates that the combined use of restraint fading and differential reinforcement is a promising treatment package in eliminating self-restraint and maintaining low levels of SIB.

Another example in which the topography of restraint was modified to facilitate fading was described by Lerman et al. (1994). The study involved a 20-year-old man with a severe intellectual disability who engaged in multiple topographies of SIB and self-restraint. Although he wore a helmet to reduce SIB, he continued to engage in various forms of self-restraint, the most common being wrapping his arms tightly inside his shirt. Because it would have been inappropriate to directly fade the size of his shirt, the researchers selected an alternative form of restraint. He was provided with a bath towel to wrap around his wrists in a manner similar to how he used his shirt. During the restraint fading process, one inch was cut from the towel whenever his SIB remained at or below predetermined levels for two consecutive sessions. Eventually, the towel was reduced to the size of a headband, and simply placing his hands beneath it significantly decreased SIB without requiring the protective helmet. Although this represented a far less restrictive form of restraint compared to his previous behavior, the ultimate goal was to eliminate restraint entirely while maintaining low levels of SIB. To accomplish this, a compliance training procedure was introduced to increase time spent out of restraint and further reduce SIB. The therapist gradually shaped movement out of restraint by delivering highly preferred edible items contingent on compliance (e.g., reaching for the edible in the absence of SIB). Over time, the requirements were systematically increased from merely reaching out for a reinforcer to completing instructional demands to access reinforcement. By the end of the study, the participant was complying with approximately 93% of instructions, and SIB occurred at near-zero rates. These results demonstrate the utility of transferring stimulus control from one topography of self-restraint to another, as well as the role of movement shaping and compliance training in increasing behavior incompatible with self-restraint while maintaining low levels of SIB.

Similar strategies to modify or replace forms of self-restraint have also been reported in the literature. Vollmer and Vorndran (1998) described a 29-year-old woman whose self-injurious behavior (SIB; self-pinching) occurred when access to a leather jacket—used as self-restraint—was restricted. Functional analysis revealed that access to the jacket reinforced SIB. Treatment involved functional communication training (FCT) in which a sign-language mand produced 30-s access to the jacket (SIB on extinction), followed by substitution of a more appropriate garment (a cardigan). Results showed substantial reductions in SIB and successful use of the substitute, illustrating how identifying and replacing restraint materials can facilitate FCT and reduce reliance on restrictive forms of self-restraint.

A more contemporary example of restraint fading was demonstrated by Banda et al. (2012). The purpose of the study was to systematically fade self-restraint (i.e., wrapping oneself in a large blanket) while simultaneously decreasing the rate of SIB in an individual diagnosed with severe autism and Tourette’s syndrome. During treatment, the blanket was gradually cut smaller over time and eventually removed. While the blanket was systematically faded, reinforcement in the form of social attention was delivered on a fixed-interval (FI) schedule, and SIB was placed on extinction. Results indicated that the intervention was effective in both reducing SIB to zero levels and eliminating the use of self-restraint, with treatment effects maintained during follow-up sessions several months later. In addition, high social validity ratings were obtained from parents and teachers, further supporting the benefits of the intervention.

There has been evidence to support the idea that for some individuals, successful interventions to reduce SIB should also indirectly weaken the maintenance of self-restraint. Silverman et al. (1984) described a single-case experiment involving a 13-year-old boy who was profoundly disabled and legally blind. His behavior was evaluated across three randomly ordered conditions: (a) no protective equipment, (b) a padded helmet, and (c) a padded helmet with padded slippers. Results showed that the padded helmet reduced head-hitting and the padded slippers reduced self-kicking. Correspondingly, use of arm and leg restraints was decreased as protective equipment was introduced. Another example of this concept was demonstrated in Fisher et al. (1996), in which physically blocking the SIB of a 19-year-old with a severe intellectual disability, thereby presumably preventing its aversive effects, reduced levels of self-restraint. An important consideration when using protective equipment to treat complex behaviors, such as severe self-injury, is the potential for novel behaviors to emerge. Powers et al. (2007) assessed the effectiveness of arm splints in reducing the occurrence of SIB, which primarily included face punching. The addition of protective equipment reduced the rates of SIB significantly; however, the subject began removing the straps that held her protective equipment on her arms and instead wrapping the straps around her fingers. This form of self-restraint not only interfered with her ability to use her hands and fingers, but it could also result in a loss of circulation to her fingers. The clinicians presented the subject with several preferred toys, which were effective in reducing the occurrence of self-restraint while maintaining low levels of SIB.

In a related manner, Kerth et al. (2009) investigated the use of clothing as a socially acceptable alternative to self-restraint for a 16-year-old boy diagnosed with autism, intermittent explosive disorder, and moderate to severe intellectual disabilities. The participant had a long history of engaging in high rates of both SIB and self-restraint. His self-restraint behaviors ranged from interlocking his hands or arms around himself or others, sitting on his hands, wrapping or twisting any rope-like material around his arms, or pulling his shirt over his head and arms. To evaluate the effects of non-contingent self-restraint, the researchers conducted a self-restraint evaluation using a reversal design. The conditions alternated between providing noncontingent access to a hooded sweatshirt and withholding access to the sweatshirt. When the participant was allowed access to the sweatshirt, SIB decreased by 52% from baseline. Moreover, when he wore the sweatshirt with the hood over his head, no other forms of self-restraint were observed. These findings suggest that systematically evaluating the relationship between SIB and self-restraint may lead to reductions in both behaviors.

The use of restraint as a consequence for periods without self-injury (e.g., differential reinforcement of other behavior; DRO) has been explored as a treatment to reduce both SIB and time spent in mechanical restraints. Favell et al. (1978) examined the effects of using physical restraint as reinforcement with three individuals with profound disabilities. All participants appeared to enjoy the mechanical devices used to prevent their self-injury (e.g., helmets, arm splints, wrist ties). During baseline sessions, all restraints were removed, and the resulting rate of SIB was recorded. Treatment sessions then introduced increasingly longer intervals in which SIB did not occur in order to access reinforcement. Reinforcement consisted of access to the individual’s preferred form of physical restraint. As the response requirement was systematically increased over the course of treatment, the duration of reinforcement periods in which the individual could access restraint was systematically decreased. Additionally, therapists attempted to minimize the overall attention provided during periods in restraint, compared to non-restraint intervals where high-quality attention and preferred items were available. Self-injury for all participants decreased to near-zero levels following the introduction of the treatment package. A subsequent experiment was conducted with one of the participants to further investigate whether self-restraint could function as a positive reinforcer. In this experiment, brief access to restraint was provided contingent upon an arbitrary response (e.g., placing a marble into a box). A reversal design was used to alternate conditions in which contingent restraint was either provided or withheld following the response. The percentage of correct responses across conditions was evaluated and was shown to increase significantly during the experimental conditions when restraint followed correct responses. Overall, the results of Favell et al. (1978) indicate that physical restraint can function as positive reinforcement for individuals with profound disabilities, and that the use of restraint contingent on the nonoccurrence of self-injury may be an effective component within a broader treatment package aimed at decreasing both SIB and time spent in restraints.

In summary, the treatment literature suggests that there is no single best self-restraint intervention, but rather a family of multicomponent, reinforcement-based packages tailored to the individual. Across studies, effective approaches typically combine (a) function-based procedures (e.g., NCR, DRA, FCT), (b) systematic fading or modification of restraint forms, and (c) access to competing or alternative stimuli that reduce both SIB and reliance on restrictive self-restraint. These packages are most successful when they prioritize reductions in SIB and restraint while simultaneously increasing adaptive engagement (e.g., task participation, toy contact, compliance). A clear implication is that clinicians should treat self-restraint within a broader, function-driven framework rather than as an isolated target, with ongoing attention to social validity and practicality for caregivers.

## 5. Conclusions

Available evidence indicates that multicomponent, reinforcement-based interventions—most often combining differential reinforcement, systematic restraint fading, and protective equipment—can reduce or eliminate self-restraint for some individuals. However, the literature is constrained by small samples and limited replication, which restrict generality. Current findings underscore substantial heterogeneity in both the form and function of self-restraint. Topographies range from self-directed postures (e.g., clasping or sitting on hands) to the use of objects, clothing, equipment, or other people to restrict movement. Functions vary across individuals—and sometimes within the same individual—encompassing numerous functional relations with SIB. This heterogeneity likely contributes to inconsistent prevalence estimates and complicates efforts to generalize assessment and treatment findings. Much of the empirical work dates to the late 20th century, and contemporary studies are relatively scarce—perhaps reflecting the complex relation between self-restraint and SIB and the practical challenges of assessment and intervention.

Clinical and research priorities include establishing standardized definitions and measurements of self-restraint topographies (with clear operational criteria, duration thresholds, and common data elements); developing mechanism-focused functional assessments that manipulate the consequences of self-restraint while holding SIB constant to distinguish shared, suppressive, and independent relations; conducting component and sequencing analyses of multicomponent treatments (e.g., DRA/FCT combined with fading and competing stimuli) to identify necessary and sufficient elements and effective schedule-thinning parameters; and evaluating generalization, maintenance, and social validity across settings, caregivers, and routines, using pre-specified outcomes such as engagement, opportunities for adaptive behavior, and caregiver feasibility. In addition, reporting standards for assessment and treatment procedures—including criteria for introducing, replacing, or fading restraint materials—would improve comparability across studies. There is also a clear role for translational research. Laboratory and human-operant models can arrange contingencies to elicit candidate functional signatures of self-restraint and test detection rules that are robust to procedural details. Coupled with computational modeling, such work could potentially generate testable predictions for clinical assessment (for example, expected changes under available versus blocked or contingent versus noncontingent access), inform treatment selection and sequencing, and accelerate the development of replicable, broadly applicable methods for assessing and treating self-restraint.

## Data Availability

This paper is a discussion article and does not include any new data. Therefore, data sharing is not applicable.

## Notes

1 It is important to note that physically self-limiting postures and repetitive self-contact behaviors also occur in the general population. Neurotypical infants and toddlers engage in hand-clasping, hand-to-mouth, or limb-holding behaviors often interpreted as self-soothing, and adults routinely adopt transient self-restricting postures (e.g., folding arms, sitting on a leg, placing hands in pockets). These forms, however, typically occur briefly, are contextually appropriate, and do not impede adaptive functioning.

2 Related behaviors have been described in nonhuman primates. In captive macaques, self-clasping—a sustained self-holding posture (e.g., forearms wrapped around the torso, hands tucked into the axillae, prolonged gripping of a limb or trunk)—is sometimes recorded alongside self-biting and other abnormal behaviors (e.g., Lutz et al., 2022; Rommeck et al., 2009). Although such postures could, in principle, constrain limb use and thereby inhibit self-injury, their functional relation to SIB has not been established experimentally; existing reports are simply descriptive. Accordingly, superficial topographic similarity should not be taken as evidence of functional homology across species but is certainly intriguing.

3 Clinical reports in Tourette syndrome describe individuals who verbally characterize their own severe body-directed behaviors—including self-injury—as involuntary. For example, Van Woert et al. (1977) describe a patient with longstanding tongue-biting and face-hitting who reported being unable to control these acts and sought assistance to prevent them. Such accounts align with the view that diminished inhibitory control can contribute to self-directed behavior, even when behavior remains sensitive to environmental contingencies.

4 Here, “experimental” denotes clinically indicated tests that vary environmental conditions (independent variables) to reveal controlling mechanisms of self-restraint; the goal is diagnostic and treatment-relevant, not “experimental” in the colloquial sense.

## Abbreviations

The following abbreviations are used in this manuscript:

SIB	Self-injurious behavior
SRC	Self-Restraint Checklist
SRQ	Self-Restraint Questionnaire
FA	Functional Analysis
PSPA	Paired-choice preference assessment
A-CSA	Augmented-competing stimulus assessment
ASRA	Alternative self-restraint assessment
NCR	Noncontingent reinforcement
DRA	Differential reinforcement of alternative behavior
FCT	Functional communication training
FI	Fixed-interval
DRO	Differential reinforcement of other behavior
